# Vaping-Associated Pneumothorax: A Systematic Review of Case Reports and Case Series

**DOI:** 10.3390/medicina61030537

**Published:** 2025-03-19

**Authors:** Moneeb Al-Taj, Alameen Alsabbah, Tariq Ma’ali, Mohammad Abu Suilik, Jehad Feras AlSamhori, Ahmad Alloubani, Ali Madha, Anita V. Goyal, Abeer Gharaibeh

**Affiliations:** 1 Faculty of Medicine, Jordan University of Science and Technology, Irbid 22110, Jordan; 2 Center for Cognition and Neuroethics, University of Michigan-Flint, Flint, MI 48502, USA; 3 Insight Research Institute, Flint, MI 48507, USA; 4 Faculty of Medicine, Yarmouk University, Irbid 21163, Jordan; 5 School of Medicine, The University of Jordan, Amman 11942, Jordan; 6 Department of Research, Insight Hospital and Medical Center, Chicago, IL 60616, USA; 7 Department of Emergency Medicine, Insight Hospital and Medical Center, Chicago, IL 60616, USA

**Keywords:** pneumothorax, spontaneous pneumothorax, vaping, electronic cigarettes, chest pain

## Abstract

*Background and Objectives*: Pneumothorax is a medical condition characterized by the accumulation of air in the pleural cavity, leading to lung collapse. While cigarette smoking is a well-known risk factor, the role of electronic cigarettes is less understood. This systematic review aimed to evaluate the outcomes of vaping-associated pneumothorax, in addition to its clinical features and management strategies, by compiling published case reports and case series. *Materials and Methods*: The choice to use case reports and case series was due to the limited availability of other types of studies on this emerging condition, as vaping-associated pneumothorax is relatively rare and primarily reported in isolated cases. Following the Preferred Reporting Items for Systematic Reviews and Meta-Analyses guidelines, we conducted a systematic search of six databases for case reports and case series. Data extraction and quality assessment were performed independently by multiple reviewers. *Results*: Seven case reports and four case series with a total number of 16 patients were included. Most patients were young, underweight men who presented with chest pain and shortness of breath. Conventional cigarette and cannabis use were commonly reported alongside vaping. The main treatment was the insertion of a chest tube, with surgical interventions reserved for severe cases. Patients who were treated non-surgically exhibited a higher recurrence rate. Additionally, specific symptoms such as chest pain radiating to the ipsilateral shoulder were associated with a higher recurrence rate. *Conclusions*: Clinicians should be vigilant for pneumothorax in at-risk individuals, consider targeted screening for symptomatic vapers, and prioritize early surgical intervention in recurrent cases to reduce complications. Further research is needed to understand the pathophysiology of vaping-associated spontaneous pneumothorax and optimal management strategies.

## 1. Introduction

Pneumothorax occurs when air accumulates in the pleural cavity, causing various degrees of lung collapse [1]. It poses a considerable challenge to healthcare systems globally, with an annual incidence of 18–28 cases per 100,000 in males and 1.2–6 cases per 100,000 in females [2].

The risk factors and underlying causes of primary spontaneous pneumothorax (PSP) include environmental influences, genetic predisposition, smoking, being an adolescent or young adult, having a thin body type, and being male [3]. Additionally, recurrent spontaneous pneumothorax occurs in 20–60% of cases, with factors such as age, gender, smoking habits, the size of the pneumothorax, a low body mass index (BMI), and the chosen treatment approach thought to influence the likelihood of recurrence [4,5]. While cigarette smoking is a well-known risk factor for primary spontaneous pneumothorax [6], little is known about the role of electronic cigarettes in its development.

The use of electronic cigarettes has risen significantly over the past decade, particularly among adolescents and young adults, with approximately 20% of high school students and 5% of adults aged 18–34 currently using them [7]. E-cigarettes function by heating a liquid to create an aerosol that is inhaled into the lungs [8]. A key appeal of e-cigarettes lies in their ability to contain various chemical substances, including nicotine, tetrahydrocannabinol (THC), and cannabinoid oils, which can be combined with additives and flavors [8].

The long-term health risks of vaping are not yet fully understood, though several case reports have linked it to negative respiratory, cardiovascular, neurological, and gastrointestinal effects [9,10,11]. Unfortunately, it is still unclear whether vaping contributes to the risk of spontaneous pneumothorax.

E-cigarette or vaping product use-associated lung injury (EVALI) is a respiratory condition that mimics other lung diseases and is diagnosed based on recent vaping history, lung infiltrates on imaging, and the exclusion of alternative causes [12]. While bleb rupture is a known cause of spontaneous pneumothorax, other vaping-related mechanisms may also contribute. Exposure to toxic aerosols, especially those containing THC and vitamin E acetate, has been linked to alveolar damage, airway inflammation, oxidative stress, and impaired lung repair, all of which may weaken lung parenchyma and increase susceptibility to barotrauma [13].

**Figure 1:**
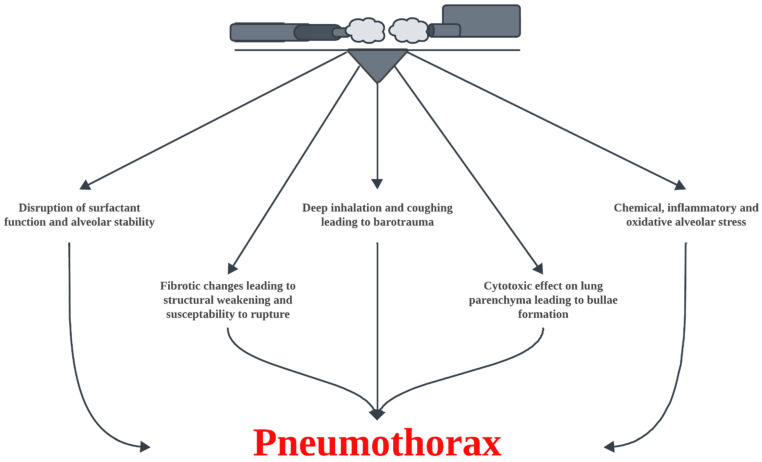
The pathophysiological mechanisms of VAP.

The pathophysiology of vaping-associated pneumothorax (VAP) is complex and not entirely understood [3]. The inhalation of chemicals such as diacetyl and 2,3-pentadione may disrupt ciliary function and trigger airway inflammation and tissue damage, leading to the formation of blebs [14]. However, the presence of intact blebs during thoracoscopy suggests additional mechanisms, such as inflammatory changes in the visceral pleura [3]. Vaping-specific breathing patterns, such as the Valsalva and Müller maneuvers, combined with the high resistance of certain vaping devices, can generate abnormal airway pressures that significantly increase the risk of pneumothorax [15,16]. Figure 1 summarizes the suggested pathophysiological mechanisms for VAP.

As VAP is a relatively rare condition with limited research studies available on it, this review aimed to systematically focus on case reports and case series on VAP. Existing studies focus on vaping-related lung injuries like EVALI but do not specifically address its link to pneumothorax. With limited data and no prior systematic reviews on this topic, understanding remains fragmented. The goal of this study is to evaluate clinical characteristics, management strategies, and outcomes of VAP, providing insights that guide clinical practice and management guidelines. With the growing popularity of vaping, particularly among teenagers and young adults, it is essential to highlight its associated risks and equip healthcare professionals with the knowledge to identify and manage this emerging health concern.

## 2. Materials and Methods

This review was conducted following the Preferred Reporting Items for Systematic Reviews and Meta-Analyses (PRISMA) guidelines, as detailed in the accompanying PRISMA checklist [17], and registered with PROSPERO (IDCRD42024603366; www.crd.york.ac.uk/prospero (accessed on 18 November 2024)).

### 2.1. Search and Selection

An electronic search of six databases, including PubMed, Scopus, Web of Science, Cochrane, Science Direct, and Google Scholar, was conducted for case reports/series regarding VAP; the search included papers published from the inception of the databases through 5 November 2024. The combination of keywords and medical subject headings we utilized included (“Vaping” OR “Vape” OR “Ecigarette” OR “E-Cigarette” OR “Ecigarettes”) AND (“Pneumothorax” OR “Spontaneous Pneumothorax” OR “Pressure Pneumothorax” OR “Tension Pneumothorax”) with the appropriate filters for each database.

Two authors participated in the study selection, beginning with the removal of duplicates using EndNote™. The screening of titles and abstracts was independently conducted by four authors using Microsoft Excel. Studies meeting the inclusion criteria were retrieved for full-text screening. Disagreements between two authors screening the same studies were resolved collaboratively. If a consensus could not be reached, a third reviewer was involved to resolve the conflict.

The inclusion criteria for this review focused on individuals diagnosed with vaping-associated pneumothorax via the assessment of clinical findings, presented in case reports or series published in peer-reviewed journals in English. Studies were included if they provided detailed information on patient demographics, vaping history, clinical presentation, diagnostic findings, management approaches, and patient outcomes. The exclusion criteria included cases in which pneumothorax was attributed to underlying lung diseases unrelated to vaping, reports that lacked detailed clinical data, or studies that focused on other vaping-related conditions, such as pneumomediastinum, without pneumothorax as a primary finding. The majority of studies were excluded as they only referred to one of the keywords or were not directly relevant to pneumothorax and vaping.

### 2.2. Data Extraction

Data extraction was performed independently by two reviewers who carefully examined the full articles and extracted relevant information from the eligible studies. The extracted data were organized into standardized Google spreadsheets. To ensure accuracy, two additional reviewers independently verified the data. The collected variables included the characteristics of both the study and participants. Study characteristics encompassed details such as the authors, title, DOI, country, number of cases, and the year of publication. Participant characteristics included demographic information (age, gender), clinical presentation and associated symptoms (e.g., chest pain, shortness of breath, coughing, and chest pain radiation), physical examination findings (e.g., vital signs, BMI), initial management strategies (e.g., oxygen supplementation, chest tube use), surgical interventions, and information about vaping and smoking history. The majority of studies selected had the outcomes and clinical characteristics of interest, but when not clearly reported in the studies, they were labeled as Not Reported (N.R), and descriptive summary statistics were only performed on the data that were available.

### 2.3. Synthesis of Results

Descriptive statistics using Microsoft Excel 2024 and Python 3.9 were utilized to analyze the extracted data, with the frequency and proportion being calculated for categorical variables. Continuous variables are reported as means ± standard deviation (SD). Logistic Regression was performed to test for associations between variables, with odds ratios, 95% confidence intervals, and statistical significance reported for each of the variables; if the variable was categorical, then the non-treatment group was used as the reference group for calculating the odds ratios.

### 2.4. Assessment of Risk of Bias

The risk of bias and quality of the included studies were assessed independently by two authors using the IHE Quality Appraisal Checklist for Case Series and the JBI Critical Appraisal Checklist for Case Reports. Any disagreements were addressed via a discussion between the authors.

## 3. Results

**Figure 2:**
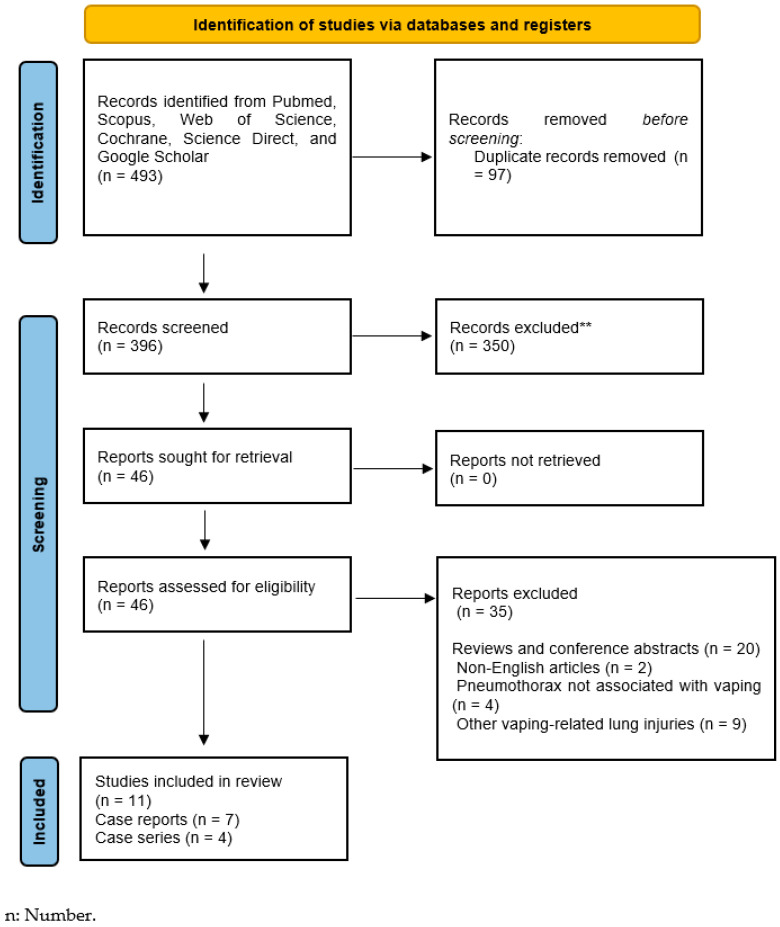
PRISMA flow diagram demonstrating the selection process and the number of studies identified, screened, excluded, and included at each stage. ** Reports were excluded after title and abstract screening.

In this systematic review, a total of 493 records were initially identified through the search strategy. After removing 97 duplicate entries, 396 records remained for screening. During the title and abstract screening process, 350 records were excluded, leaving 46 articles for full-text review. Of these, 35 articles did not meet the inclusion criteria and were subsequently excluded. Ultimately, 11 articles were included in the review, comprising 7 case reports and 4 case series (Figure 2).

All of the included studies were published within the last 6 years, with the majority conducted in the USA. This study encompassed case reports and case series. A summary of the included studies and key patient characteristics is provided in Table 1. The quality assessment and overall evaluation of the included articles are presented in the Supplementary Materials.

The majority of patients (81%) were young men, predominantly classified as underweight (BMI < 18.5) or at the lower end of the normal weight range, as indicated in Table 2. The patients primarily complained of chest pain or shortness of breath, while chest pain radiating to the ipsilateral shoulder was noted in approximately one-third of cases, and coughing was a presenting symptom in half of the patients. Some presented with atypical symptoms, such as abdominal pain, extremity tingling, or a sense of impending doom. Conventional cigarette smoking was documented in 25% of cases, with a higher prevalence of cannabis use. Among 10 cases with recorded heart rates, the average was 87 beats per minute, with tachycardia (>100 bpm) observed in three patients. Evidence of tension physiology, such as mediastinal shift, increased intercostal spaces, and diaphragmatic depression, was noted in six cases, while blebs or bullae were identified in over half.

Most cases of VAP were initially managed with the insertion of a chest tube, while oxygen supplementation was used less frequently. Needle aspiration was performed in only one case. Among the 16 patients included in the study, 10 underwent surgical intervention, which was performed using video-assisted thoracoscopic surgery (VATS). Blebectomy and pleurodesis were each performed in seven cases. Of the pleurodesis procedures, five were mechanical, while the remaining two were chemical. Pleurectomy was conducted less frequently, being performed in four patients.

The recurrence of pneumothorax was observed in six cases following the initial presentation, with all of these cases being managed non-surgically beforehand. The average interval between episodes was approximately two weeks. Patients presenting with chest pain radiating to the ipsilateral shoulder were significantly more likely to experience recurrent pneumothorax (Table 3).

Parenchymal abnormalities in the lung on imaging, characteristic of the e-cigarette or EVALI outbreak, were reported in six cases; these included ground-glass opacities, crazy paving patterns, large bullae, bronchiectasis, and lung volume loss. Parenchymal abnormalities in vaping-associated pneumothorax were significantly linked to age and the presentation of a cough (Table 4). Other factors such as a mediastinal shift and surgical needs showed high but non-significant odds.

No variables reached statistically significant associations with the presence of bullae/blebs in vaping-associated pneumothorax cases. However, the presence of tension had a higher odds ratio (OR 7.5, 95% CI: 0.6–92.3, *p* = 0.091), as shown in Table 3.

Cannabis users were significantly more likely to present with radiating chest pain to the ipsilateral shoulder (Table 4). They were also more likely to require oxygen supplementation and to show evidence of tension and lung parenchymal abnormalities on imaging, but these associations were not statistically significant.

## 4. Discussion

The results demonstrate a predominant prevalence among men; this aligns with the existing literature, which shows that young men are at a higher risk of spontaneous pneumothorax [29]. A higher prevalence of smoking, an earlier initiation of smoking, and smaller airways, which can lead to earlier airway obstruction, are potential explanations for this finding [6,30].

Given the limited case reports and series available, sensitivity analysis was not needed for most of the variables, as they were statistically insignificant. For the significant variables, the ORs fluctuated based on the studies included. Furthermore, as the sample size was small, the variance inflation factor was unable to be calculated to test for covariance. As most studies were case studies, each cell had <5 counts and statistical tests for heterogeneity were unable to be conducted.

The mean BMI in our study was 18.93 ± 2.88, suggesting that a significant proportion of the patients were underweight. This aligns with evidence showing that a lower BMI is associated with a higher risk of pneumothorax [31]. A leaner physique, characterized by a thinner chest wall and reduced subcutaneous fat, may provide less resistance to sudden increases in lung pressure, potentially leading to pleural damage and the development of pneumothorax [32].

Mediastinal shift was observed in 37.5% of vaping-associated pneumothorax cases, a frequency notably higher than the 17% reported in a study analyzing 176 spontaneous pneumothorax presentations [33]. This discrepancy may be attributed to rapid and severe changes in the pleural pressure induced by vaping.

Blebs or bullae were identified in 56.3% of vaping-associated pneumothorax cases, compared to 73.3% reported in a study on general pneumothorax cases [34]. In spontaneous pneumothorax, these structural abnormalities are a common cause, particularly in primary cases where they result from congenital or acquired weaknesses in the lung parenchyma [35]. The slightly lower prevalence of blebs or bullae in vaping-associated cases may suggest distinct underlying mechanisms, such as acute alveolar damage, inflammation, or chemical toxicity caused by vaping aerosols, which can cause pneumothorax without pre-existing structural defects.

In our study, 75% of VAP cases were managed with the insertion of a chest tube, while needle aspiration was used in one case. Oxygen supplementation was provided in 56.3% (9/16) of cases, and 1 case was managed through observation alone. These management patterns are consistent with another study reporting similar approaches to PSP [34]. Nonetheless, research indicates that needle aspiration can reduce patients’ length of hospitalization and rates without significantly impacting outcomes such as recurrence or complications when compared to chest tube insertion in primary spontaneous pneumothorax [36]. The limited use of needle aspiration in our study may be attributed to the larger pneumothorax size often observed in vaping-associated cases, where chest tube insertion is typically preferred for initial management. However, aspiration puncture has also been recognized as an effective treatment option, particularly in regions where it is more frequently practiced.

Among the 16 patients included in our study, 10 underwent surgical intervention, all via VATS. This is consistent with other studies, which identify it as the preferred surgical modality for PSP due to its safety, reduced hospital stays, and improved patient satisfaction [37,38]. Notably, no recurrences were reported among the patients who underwent VATS, aligning with evidence showing that preventive surgical approaches significantly lower recurrence rates compared to non-preventive methods such as observation, needle aspiration, or chest drainage [39]. These findings underscore the role of VATS as a definitive treatment for PSP, delivering durable outcomes and an enhanced quality of care.

Pleurodesis was performed in seven cases, with five being mechanical and two chemical. Evidence from other studies suggests comparable outcomes between mechanical and chemical pleurodesis, although some findings indicate a lower recurrence rate with talc chemical pleurodesis compared to mechanical methods [40]. One study demonstrated statistical significance favoring talc, while a larger, more recent study found no significant difference in recurrence over a 10-year follow-up [41]. Further research is needed to confirm the most effective intervention.

Six cases experienced recurrence after the initial presentation, with all of them initially managed using non-surgical methods. This corresponds with the recurrence rates reported in previous studies, with rates of 34% in patients treated with chest tubes compared to rates of 12% in those undergoing VATS [42]. While international guidelines generally recommend surgery only in certain situations [43], the increasing preference for early surgical intervention in some cases suggests a potential shift in the standard of care due to its effectiveness in reducing recurrence. However, the optimal approach remains controversial, requiring individualized decision-making based on clinical factors and patient preferences.

Chest pain radiating to the ipsilateral shoulder was significantly associated with recurrence, while coughing showed a protective effect. These findings complement the existing literature, where radiographic evidence of blebs was inconsistently associated with the risk of recurrence in prior studies [35,36,42,43,44]; in addition, female sex was linked to increased recurrence in a systematic review [45]. Additionally, smoking cessation has been shown to reduce the risk of recurrence four-fold [45], highlighting the importance of addressing modifiable factors that affect the risk of recurrence. The variability in associations underscores the multifactorial nature of pneumothorax recurrence, suggesting that both clinical and demographic factors should guide management and recurrence prevention strategies.

A significant proportion of cases exhibited parenchymal abnormalities in the lung, including ground-glass opacities, “crazy paving” patterns, and the presence of unusual large bullae. These findings are consistent with descriptions of lung injury in the EVALI outbreak [46], suggesting a potential overlap in pathology between EVALI and pneumothorax in vape users. Notably, older patients had significantly higher odds of presenting with parenchymal abnormalities, which may indicate the cumulative effect of vaping over time.

EVALI shares clinical features with PSP, such as chest pain, shortness of breath, and imaging findings like ground-glass opacities. While PSP is linked to bleb formation, vaping-related pneumothorax may also involve inflammation and chemical toxicity, highlighting the possible shared pathophysiology [46,47].

Our study found that the use of cannabis was prevalent among VAP cases, with mediastinal shift frequently observed. This aligns with prior evidence showing that cannabis smokers are more prone to severe respiratory symptoms, bullous lung disease, and tension pneumothorax physiology [48], highlighting their higher risk profile and need for targeted management.

Compared to traditional smoking, vaping has been associated with higher airway reactivity, increased deposition of ultrafine particles, and lipid-laden macrophages, all of which may contribute to lung instability and pneumothorax. Additionally, emerging evidence suggests that vaping-related changes in lung compliance and microvascular injury may exacerbate ventilation–perfusion mismatch, further differentiating its effects from conventional smoking [49].

Given the potential link between vaping and pneumothorax, clinicians should maintain a high index of suspicion for pneumothorax in patients with vaping history presenting with chest pain or respiratory symptoms. Screening for vaping history in young patients with spontaneous pneumothorax may aid in the early identification of at-risk individuals. Additionally, our study supports the growing preference for early surgical intervention, particularly VATS, in recurrent cases due to its lower recurrence rates and improved outcomes. While further research is needed to establish causation, raising clinical awareness of this association can help guide timely diagnosis and optimal management strategies.

## 5. Strengths and Limitations

This study highlights concerns regarding the potential risks associated with vaping. A broad search of multiple databases ensures a comprehensive collection of case reports and series. These studies were assessed for their quality and risk of bias, making the results more reliable. This review provides a detailed analysis of patient characteristics, management strategies, and the outcomes of VAP, thus offering valuable clinical insights.

This study has several limitations, primarily its reliance on case reports and case series, which are subject to publication and recall bias, as well as missing data. The retrospective nature and small sample size limit causation analysis and generalizability, especially given the under-reporting of vaping in clinical settings. Additionally, some patients had a history of smoking or cannabis use, making it difficult to isolate vaping as an independent risk factor. While this review identifies clinical patterns, it does not determine whether VAP differs from spontaneous cases in non-vapers. Future prospective studies with standardized data collection, imaging, and histological analysis are needed to better understand this association.

## 6. Conclusions

This systematic review highlights that pneumothorax in individuals who vape mainly affects young underweight men, especially those who use cannabis. The most common presentation included chest pain and shortness of breath. Atypical presentations such as abdominal pain were reported. On imaging, some common parenchymal abnormalities in the lung were found to be more prevalent, such as ground-glass opacities and bullae. The management of pneumothorax typically involves the insertion of a chest tube, while surgical intervention is reserved for severe cases. The higher rate of recurrence in patients presenting with chest pain radiating to the ipsilateral shoulder was a particularly interesting association. Despite tension physiology being linked to VAP, no significant associations with the presence of blebs were found.

With the rising prevalence of vaping, clinicians should maintain a high index of suspicion for pneumothorax in at-risk individuals and consider targeted screening for symptomatic vapers. Early surgical intervention may be preferable, particularly in recurrent cases, to reduce long-term complications. Future research should aim to identify high-risk populations and refine management strategies to minimize recurrence rates.

## Figures and Tables

**Table 1:** Summary of key patient characteristics and design by studies included.

Author (ID)	Study Design	Study N	Country	Chest Drain	Recurrence	Surgical Intervention	Age (Mean, SD)	Gender, Male N (%)	BMI (Mean, SD)	Radiation to the Ipsilateral Shoulder	Coughing	Smoking	Cannabis	Lung Parenchymal Abnormalities	Evidence of Tension	Bleb/Bullae	Oxygen	Chest Drain	Recurrence	Surgical Intervention
Skertich et al., 2019 [18]	Case series	2	USA	Yes	Yes	Yes	15.5 (0.5)	2 (100%)	18.8 (0.20)	0 (0%)	0 (0%)	0 (0%)	0 (0%)	0 (0%)	0 (0%)	1 (50%)	0 (0%)	2 (100%)	2 (100%)	2 (100%)
Ali et al., 2020 [19]	Case series	2	USA	Yes	No	Yes	23.0 (11.0)	2 (100%)	NA	0 (0%)	2 (100%)	0 (0%)	0 (0%)	1 (50%)	1 (50%)	1 (50%)	0 (0%)	2 (100%)	0 (0%)	1 (50%)
Wieckowska et al., 2021 [20]	Case report	1	USA	No	No	No	19 (NA)	1 (100%)	20.5 (NA)	0 (0%)	0 (0%)	1 (100%)	1 (100%)	0 (0%)	0 (0%)	0 (0%)	1 (100%)	0 (0%)	0 (0%)	0 (0%)
Borchert et al., 2021 [21]	Case report	1	Germany	Yes	No	Yes	34 (NA)	1 (100%)	23.6 (NA)	0 (0%)	1 (100%)	1 (100%)	0 (0%)	0 (0%)	0 (0%)	1 (100%)	1 (100%)	1 (100%)	0 (0%)	1 (100%)
Ashraf et al., 2021 [22]	Case series	4	USA	No	No	No	23.0 (7.0)	3 (75%)	18.6 (3.12)	3 (75%)	2 (50%)	1 (25%)	4 (100%)	2 (50%)	0 (0%)	3 (75%)	2 (50%)	0 (0%)	0 (0%)	0 (0%)
Bonilla et al., 2019 [23]	Case report	1	USA	Yes	Yes	No	18 (NA)	1 (100%)	16.9 (NA)	1 (100%)	0 (0%)	0 (0%)	1 (100%)	0 (0%)	1 (100%)	1 (100%)	0 (0%)	1 (100%)	1 (100%)	0 (0%)
Sharma et al., 2019 [24]	Case report	1	USA	Yes	No	Yes	35 (NA)	1 (100%)	NA	0 (0%)	1 (100%)	0 (0%)	1 (100%)	1 (100%)	1 (100%)	1 (100%)	1 (100%)	1 (100%)	0 (0%)	1 (100%)
Deskins et al., 2022 [25]	Case report	1	USA	Yes	Yes	Yes	15 (NA)	0 (0%)	NA	1 (100%)	0 (0%)	0 (0%)	1 (100%)	0 (0%)	0 (0%)	1 (100%)	1 (100%)	1 (100%)	1 (100%)	1 (100%)
Wu & Mohammed, 2020 [26]	Case report	1	USA	No	No	Yes	35 (NA)	1 (100%)	NA	0 (0%)	1 (100%)	0 (0%)	1 (100%)	1 (100%)	0 (0%)	0 (0%)	1 (100%)	0 (0%)	0 (0%)	1 (100%)
Reddy et al., 2020 [27]	Case series	1	USA	No	No	No	17 (NA)	0 (0%)	NA	0 (0%)	1 (100%)	0 (0%)	1 (100%)	1 (100%)	0 (0%)	0 (0%)	1 (100%)	0 (0%)	0 (0%)	0 (0%)
Khuan & Wong, 2023 [28]	Case report	1	Malaysia	Yes	No	No	24 (NA)	1 (100%)	16.3 (NA)	0 (0%)	1 (100%)	1 (100%)	0 (0%)	0 (0%)	0 (0%)	0 (0%)	1 (100%)	1 (100%)	0 (0%)	0 (0%)

BMI: body mass index.

**Table 2:** Descriptive analysis of clinical parameters, findings, and treatment approaches.

Variable	Category	N (%)	Mean, SD
Age	15–25	12 (75)	23.44 (8.1)
	26–35	4 (25)	
Gender	Male	13 (81)	
BMI	Normal	4 (40)	18.93 (2.88)
	Underweight	6 (60)	
Side of pneumothorax	Right	8 (50)	
	Left	6 (37.5)	
	Bilateral	2 (12.5)	
Radiation of chest pain		5 (31.3)	
Coughing		9 (56.3)	
Smoking		4 (25)	
THC		10 (62.5)	
Heart rate	Normal	7 (70)	87.1
	Tachycardia	3 (30)	
Respiratory rate	Normal	6 (67)	20
	Tachypnea	3 (33)	
Oxygen	≥95%	11 (92)	
Lung parenchymal abnormalities		6 (37.5)	
Bleb/bullae		9 (56.3)	
Evidence of tension		6 (37.5)	
Observation		1 (6.25)	
Needle aspiration		1 (6.25)	
Oxygen		9 (56.3)	
Chest tube		12 (75)	
Recurrence		6 (37.5)	
Surgical intervention		10 (62.5)	

BMI: body mass index. THC: tetrahydrocannabinol (cannabis).

**Table 3:** The association of clinical parameters, findings, and treatment approaches in relation to the presence of bullae/blebs and recurrence of pneumothorax.

	Bullae/Bleb (N%)			Recurrence N (%)		
		OR (95% CI)	*p*-Value		OR (95% CI)	*p*-Value
Age		4.800 (0.397–58.013)	0.197		0.300 (0.025–3.626)	0.33
26–35	4 (80%)			1 (20)		
15–25 ^R^	5 (45.5)			5 (45.5)		
Gender		0.583 (0.042–8.146)	0.687		1.250 (0.089–17.653)	0.869
Male	7 (53.8)			5 (38.5)		
RIS		4.800 (0.397–58.013)	0.197		18.000 (1.242–260.918)	0.018 *
	4 (80)			4 (80)		
Coughing		0.938 (0.128–6.875)	0.949		0.050 (0.004–0.706)	0.013 *
	5 (55.6)			1 (11.1)		
Oxygen usage		0.938 (0.128–6.875)	0.949		0.214 (0.024–1.877)	0.152
	5 (55.6)			2 (22.2)		
Chest tube		6.000 (0.463–77.750)	0.146		.	0.074
	8 (66.7)			6 (50)		
SI		4.667 (0.533–40.886)	0.152		0.200 (0.017–2.386)	0.182
	7 (70)			1 (16.7)		
LPA		2.000 (0.244–16.362)	0.515		2.333 (0.287–18.965)	0.424
	4 (66.7)			3 (50)		
ET		7.500 (0.621–90.646)	0.091		2.000 (0.244–16.362)	0.515
	5 (83.3)			4 (44.4)		
Recurrence		2.000 (0.244–16.362)	0.515		0.467 (0.037–5.903)	0.551
	4 (66.7)			1 (25)		
Smoking		0.714 (0.074–6.922)	0.771		1.333 (0.161–11.075)	0.79
	2 (50)			4 (40)		
THC		1.500 (0.195–11.536)	0.696			
	6 (60)					

OR: odds ratio. CI: confidence interval. ^R^: reference group. *: significant finding. RIS: radiation to the ipsilateral shoulder. SI: surgical intervention. LPA: lung parenchymal abnormality. ET: evidence of tension. THC: tetrahydrocannabinol (cannabis).

**Table 4:** The association of clinical parameters, findings, and treatment approaches in relation to the presence of lung parenchymal abnormalities and THC consumption.

	Lung Parenchymal Abnormalities N (%)			THC N (%)		
		OR (95% CI)	*p*-Value		OR (95% CI)	*p*-Value
Age		18.000 (1.242–260.918)	0.018 *		0.857 (0.098–7.510)	0.889
26–35	4 (80)			3 (60)		
15–25 ^R^	2 (18.2)			7 (63.6)		
Gender		0.222 (0.015–3.221)	0.247		.	0.137
Male	4 (30.8)			7 (53.8)		
RIS		0.300 (0.025–3.626)	0.33		.	0.037 *
Yes	1 (20)			5 (100)		
Coughing		.	0.006 *		0.500 (0.061–4.091)	0.515
Yes	6 (66.7)			5 (55.6)		
Oxygen usage		2.000 (0.244–16.362)	0.515		4.667 (0.533–40.886)	0.152
Yes	4 (44.4)			7 (77.8)		
Chest tube		0.500 (0.050–4.978)	0.551		.	0.074
Yes	4 (33.3)			6 (50)		
SI		5.000 (0.419–59.657)	0.182		0.750 (0.090–6.230)	0.79
Yes	5 (50)			6 (60)		
ET		8.000 (0.803–79.655)	0.062		5.000 (0.419–59.657)	0.182
Yes	4 (66.7)			5 (83.3)		
Bullae/Bleb		2.000 (0.244–16.362)	0.515		5.000 (0.419–59.657)	0.182
Yes	4 (44.4)			5 (83.3)		
Recurrence		0.200 (0.017–2.386)	0.182		1.500 (0.195–11.536)	0.696
Yes	1 (16.7)			6 (66.7)		
Smoking		0.467 (0.037–5.903)	0.551		1.333 (0.161–11.075)	0.79
Yes	1 (25)			4 (66.7)		
THC		5.000 (0.419–59.657)	0.182			
Yes	5 (50)					

OR: odds ratio. CI: confidence interval. ^R^: reference group. *: significant finding. RIS: radiation to the ipsilateral shoulder. SI: surgical intervention. ET: evidence of tension. THC: tetrahydrocannabinol (cannabis).

## Data Availability

The data presented in this study are available within this manuscript.

## Supplementary Materials

The following supporting information can be downloaded at: https://www.mdpi.com/article/10.3390/medicina61030537/s1, Table S1: The quality assessment and overall appraisal of included case reports.; Table S2: The quality assessment and overall appraisal of the included case series.
