# Supplementary material for: Vaping-Associated Pneumothorax: A Systematic Review of Case Reports and Case Series
Source: Medicina (Kaunas). 2025 Mar 19;61(3):537. doi: 10.3390/medicina61030537 (PMC11943585; doi:10.3390/medicina61030537)
Supplement: Supplementary file 1 [file medicina-61-00537-s001.zip › medicina-3488296-supplementary.pdf]

Supplementary Table S1. The quality assessment and overall appraisal of included case reports.

ID	Country	Study design	Were patient's demographic characteristics clearly described?(Yes,No, Unclear, Not applicable)	Was the patient's history clearly described and presented as a timeline?(Yes,No, Unclear, Not applicable)	Was the current clinical condition of the patient on presentation clearly described?(Yes,No, Unclear, Not applicable)	Were diagnostic tests or assessment methods and the results clearly described?(Yes,No, Unclear, Not applicable)	Was the intervention(s) or treatment procedure(s) clearly described?(Yes,No, Unclear, Not applicable)	Was the post-intervention clinical condition clearly described?(Yes,No, Unclear, Not applicable)	Were adverse events (harms) or unanticipated events identified and described?(Yes,No, Unclear, Not applicable)	Does the case report provide takeaway lessons?(Yes,No, Unclear, Not applicable)	Overall appraisal: (Include, Exclude, Seek further info)
[1]	USA	Case Report	Yes	Yes	Yes	Yes	Yes	Yes	No	Yes	Include
[2]	Germany	Case Report	Yes	Yes	Yes	Yes	Yes	Yes	No	Yes	Include
[3]	USA	Case Report	Yes	Yes	Yes	Yes	Not applicable	Not applicable	No	unclear	Include
[4]	USA	Case Report	Yes	Yes	Yes	Yes	Yes	unclear	No	Yes	Include
[5]	USA	Case Report	Yes	Yes	Yes	Yes	Yes	unclear	No	Yes	Include
[6]	USA	Case Report	No	unclear	No	Yes	Yes	No	No	Yes	exclude
[7]	Malaysia	Case Report	Yes	Yes	Yes	Yes	Yes	Yes	No	Yes	Include

Supplementary Table S2. The quality assessment and overall appraisal of the included case series.

ID	Country	Study design	Was the hypothesis/aim/objective of the study clearly stated? (Yes,Partial,No)	Was the study conducted prospectively? (Yes,unclear, No)	Were the cases collected in more than one centre? (Yes,unclear, No)	Were patients recruited consecutively? (Yes,unclear, No)	Were the characteristics of the patients included in the study described? (Yes,Partial, No)	Were the eligibility criteria (i.e. inclusion and exclusion criteria) for entry into the study clearly stated? (Yes,Partial, No)	Did patients enter the study at a similar point in the disease?(Yes,unclear,No)	Was the intervention of interest clearly described? (Yes,Partial, No)	Were additional interventions (co-interventions) clearly described? (Yes,Partial, No)	Were relevant outcome measures established a priori? (Yes,Partial, No)	Were outcome assessors blinded to the intervention that patients received? (Yes,unclear, No)	Were the relevant outcomes measured using appropriate objective/subjective methods? (Yes,Partial, No)	Were the relevant outcome measures made before and after the intervention? (Yes, unclear, No)	Were the statistical tests used to assess the relevant outcomes appropriate? (Yes, unclear, No)	Was follow-up long enough for important events and outcomes to occur? (Yes, unclear, No)	Were losses to follow-up reported? (Yes, unclear, No)	Did the study provided estimates of random variability in the data analysis of relevant outcomes? (Yes, Partial,No)	Were the adverse events reported? (Yes, Partial,No)	Were the conclusions of the study supported by results? (Yes, unclear, No)	Were both competing interests and sources of support for the study reported? (Yes, Partial,No)
[8]	USA	Case Series	Yes	No	No	Unclear	Yes	No	Yes	Yes	Yes	No	No	Yes	No	Unclear	Yes	No	No	No	Yes	Yes
[9]	USA	Case Series	Yes	No	No	Unclear	Yes	No	Unclear	Yes	Unclear	No	No	Yes	No	Unclear	Unclear	No	No	No	Yes	Yes

**[10]**	USA	Case Series	Yes	No	Unclear	Unclear	Yes	No	Yes	Yes	Yes	No	No	Yes	No	Unclear	Yes	No	No	No	Yes	Yes
**[11]**	**USA**	**Case Series**	**Yes**	**No**	**No**	**Yes**	**Yes**	**Yes**	**Yes**	**Yes**	**Yes**	**No**	**No**	**Yes**	**No**	**Unclear**	**Unclear**	**No**	**No**	**No**	**Yes**	**Yes**

References:

[1] Wieckowska J, Assaad U, Aboudan M. Pneumothorax secondary to vaping. Respir Med Case Rep. 2021 May 5;33:101421. doi: 10.1016/j.rmcr.2021.101421. PMID: 34401268; PMCID: PMC8348149.

[2] Borchert DH, Kelm H, Morean M, Tannapfel A. Reporting of pneumothorax in association with vaping devices and electronic cigarettes. BMJ Case Rep. 2021 Dec 17;14(12):e247844. doi: 10.1136/bcr-2021-247844. PMID: 34920999; PMCID: PMC8685946.

[3] Bonilla A, Blair AJ, Alamro SM, Ward RA, Feldman MB, Dutko RA, Karagounis TK, Johnson AL, Folch EE, Vyas JM. Recurrent spontaneous pneumothoraces and vaping in an 18-year-old man: a case report and review of the literature. J Med Case Rep. 2019 Sep 9;13(1):283. doi: 10.1186/s13256-019-2215-4. PMID: 31495337; PMCID: PMC6732835.

[4] Sharma M, Anjum H, Bulathsinghala CP, Buch M, Surani SR. A Case Report of Secondary Spontaneous Pneumothorax Induced by Vape. Cureus. 2019 Nov 4;11(11):e6067. doi: 10.7759/cureus.6067. PMID: 31827996; PMCID: PMC6890157.

[5] Deskins SJ, Luketich SK, Al-Qatarneh S. Recurrent spontaneous pneumothorax in a 15-year-old female associated with electronic cigarettes. Pediatr Pulmonol. 2022 Oct;57(10):2568-2570. doi: 10.1002/ppul.26058. Epub 2022 Jul 29. PMID: 35856236.

[6] Wu M, Mohammed TH. Electronic Cigarette or Vaping Product Use-associated Lung Injury: Diffuse Alveolar Damage. Radiol Cardiothorac Imaging. 2020 Apr 2;2(2):e200027. doi: 10.1148/ryct.2020200027. PMID: 33778558; PMCID: PMC7978002.

[7] Khuan WH, Wong CK. Vaping - A Precipitating Factor of Pneumothorax in A Smoker. Malaysian Journal of Medicine and Health Sciences (2023) 19(1):366-368. doi:10.47836/mjmhs19.1.46

[8] Skertich NJ, Sullivan GA, Madonna MB, Shah AN.. Journal of Pediatric Surgery Case Reports. Elsevier; 50:101305. https://doi.org/10.1016/j.epsc.2019.101305

[9] Ali M, Khan K, Buch M, Ramos-Ramirez M, Sharma M, Patel S, Choudhury S, Anjum H, Khan A, Surani S. A Case Series of Vaping-Induced Lung Injury in a Community Hospital Setting. Case Rep Pulmonol. 2020 Jan 31;2020:9631916. doi: 10.1155/2020/9631916. PMID: 32082682; PMCID: PMC7013298.

[10] Ashraf O, Nasrullah A, Karna R, Alhajhusain A. Vaping associated spontaneous pneumothorax - A case series of an enigmatic entity! Respir Med Case Rep. 2021 Oct 23;34:101535. doi: 10.1016/j.rmcr.2021.101535. PMID: 34745871; PMCID: PMC8551642.

[11] Reddy A, Jenssen BP, Chidambaram A, Yehya N, Lindell RB. Characterizing e-cigarette vaping-associated lung injury in the pediatric intensive care unit. Pediatr Pulmonol. 2021 Jan;56(1):162-170. doi: 10.1002/ppul.25086. Epub 2020 Oct 7. PMID: 32970368; PMCID: PMC8008480.
